# ERK1 Regulates the Hematopoietic Stem Cell Niches

**DOI:** 10.1371/journal.pone.0030788

**Published:** 2012-01-30

**Authors:** Nathalie Saulnier, Soizic Guihard, Xavier Holy, Elodie Decembre, Pierre Jurdic, Denis Clay, Vincent Feuillet, Gilles Pagès, Jacques Pouysségur, Françoise Porteu, Murielle Gaudry

**Affiliations:** 1 Institut Cochin, Université Paris Descartes, Sorbonne Paris Descartes, CNRS (UMR 8104), Paris, France; 2 Inserm U1016, Paris, France; 3 Service histologie et réparation tissulaire, IRBA/IMASSA, Brétigny-sur-Orge, France; 4 Institut de Génomique Fonctionnelle de Lyon, Université de Lyon, Université Lyon 1, CNRS, INRA, Ecole Normale Supérieure de Lyon, Lyon, France; 5 Inserm U972, Institut André Lwoff, Hôpital Paul Brousse, Villejuif, France; 6 Institut de recherche Signalisation, Biologie du Développement et Cancer, Université de Nice, France; Instituto Nacional de Câncer, Brazil

## Abstract

The mitogen-activated protein kinases (MAPK) ERK1 and ERK2 are among the major signal transduction molecules but little is known about their specific functions *in vivo*. ERK activity is provided by two isoforms, ERK1 and ERK2, which are ubiquitously expressed and share activators and substrates. However, there are not *in vivo* studies which have reported a role for ERK1 or ERK2 in HSCs and the bone marrow microenvironment. The present study shows that the ERK1-deficient mice present a mild osteopetrosis phenotype. The lodging and the homing abilities of the ERK1^−/−^ HSC are impaired, suggesting that the ERK1^−/−^-defective environment may affect the engrafment of HSCs. Serial transplantations demonstrate that ERK1 is involved in the maintenance of an appropriate medullar microenvironment, but that the intrinsic properties of HSCs are not altered by the ERK1^−/−^ defective microenvironment. Deletion of ERK1 impaired *in vitro* and *in vivo* osteoclastogenesis while osteoblasts were unaffected. As osteoclasts derive from precursors of the monocyte/macrophage lineage, investigation of the monocytic compartment was performed. *In vivo* analysis of the myeloid lineage progenitors revealed that the frequency of CMPs increased by approximately 1.3-fold, while the frequency of GMPs significantly decreased by almost 2-fold, compared with the respective WT compartments. The overall mononuclear-phagocyte lineage development was compromised in these mice due to a reduced expression of the M-CSF receptor on myeloid progenitors. These results show that the cellular targets of ERK1 are M-CSFR-responsive cells, upstream to osteoclasts. While ERK1 is well known to be activated by M-CSF, the present results are the first to point out an ERK1-dependent M-CSFR regulation on hematopoietic progenitors. This study reinforces the hypothesis of an active cross-talk between HSCs, their progeny and bone cells in the maintenance of the homeostasis of these compartments.

## Introduction

Adult hematopoietic stem cells (HSCs) exist in a relatively quiescent state in the bone marrow (BM) microenvironment to execute long-term self renewal and multilineage differentiation functions [1], [2]. The maintenance of HSC quiescence involves both intrinsic and extrinsic mechanisms. HSCs interact with the BM microenvironment in specific anatomical and functional areas, referred as niches, a microenvironment of different cell types and extracellular matrix molecules that dictate stem cell self-renewal and progeny production *in vivo* [3]. Many studies have begun to define the medullar niche but few data are available concerning the cells and factors supporting HSCs in those niches. Likewise, the signaling pathways governing activation of various stromal cells and the specific relationship between this activation state and expansion/maintenance of HSCs are largely unexplored. HSCs reside in medullar niches, mainly localized in the endosteum. The so-called endosteal stem cell niche is a dynamically remodeled tissue that allows interactions between stem cells and their partners, including osteoblasts and stromal cells, which provide HSCs with signals for their homeostatic quiescent state [4], [5], [6], [7]. The architecture of the HSC niche is tightly controlled by a balance between the bone-forming cells (osteoblasts), and the bone-resorbing cells (osteoclasts) [8].

While osteoblasts derive from mesenchymal cells, osteoclasts derive from mononuclear precursors of the monocytic/macrophage lineage [9]. Monocytes develop from hematopoietic stem cells in the bone marrow through several intermediate progenitors that restrict progressively their differentiation potential [10]. The prevalent model for myeloid commitment implicates that progenitors pass through the common myeloid progenitor (CMP), the granulocyte/macrophage progenitor (GMP), and the macrophage/dendritic cell progenitors (MDP) stages [11]. Each of these steps is tightly regulated by the interplay between cytokine-induced signaling pathway and transcription factor activity. Monocyte-colony stimulating factor (M-CSF), through its binding to M-CSFR (CD115, Csf1-R) [12] plays an essential role in both proliferation and differentiation of the myeloid progenitors. M-CSF, and receptor activator of nuclear factor kappa B ligand (RANKL), are essential for osteoclastogenesis [9], [13], [14], [15], [16]. Mutational inactivation or genetic ablation of these molecules results in a lack of osteoclast differentiation and consequent defects in BM cavity formation, a condition termed osteopetrosis in which BM cavities are filled with bone [17], [18], [19]. Accumulating evidence suggests that osteoclasts induce hematopoietic activity by secreting bone-resorbing enzymes which promote the mobilization of HSCs from their quiescent state [7]. However, the influence of HSCs on their microenvironment is not well understood. A recent report demonstrated a cross-talk between HSCs and bone cells in the maintenance of both hematopoietic and bone homeostasis [20].

The ERK MAPK kinases are major signaling actors relaying signals elicited by cytokines, morphogens or integrins. The MAPK pathway exerts pleiotropic effects on cell cycle, apoptosis and differentiation, and has been implicated in the control of many cellular responses. ERK activity is provided by two isoforms, named ERK1 and ERK2, that are highly similar, ubiquitously expressed and that share activators and substrates. We and others have found that the ERK pathway is specifically required for the differentiation of various hematopoietic lineages [21], [22], [23], [24]. However, no studies have reported the role of ERK1 or ERK2 in HSCs *in vivo*. Gene disruption has suggested specific functions for each isoform. Ablation of ERK2 induces embryonic lethality with death of the animals *in utero* at day 7.5, due to defects in the mesoderm and placental development [25]. Conversely, ERK1^−/−^ mice are viable, fertile and present minor defects, showing that ERK1 is dispensable for mouse development and could be compensated by ERK2. So far, ERK1 has been shown to be specifically required for *in vitro* and *in vivo* adipogenesis, terminal differentiation of T lymphocytes, long-term memory and erythropoiesis [26], [27], [28], [29].

The involvement of the MAPK pathway in myeloid maturation has been implicated in several myeloid cell lines [30]. Using ERK chemical inhibitors, Hsu et al. have shown that ERK signaling is involved in the early myeloid commitment of HSCs [31]. On the other hand, ERK inhibitors have been reported to alter osteoclast differentiation and function [32]. Very recently, He Y et al. reported the preponderant role of ERK1 over ERK2 isoform in controlling osteoclast differentiation and bone resorption activity [33]. This suggests that ERKs might have a role in stem cell niche regulation. However, this possibility together with the precise role of the ERK pathway in the regulation of myelopoieseis *in vivo*, to our knowledge, has not been reported.

We show here that ERK1-deficient mice present an alteration of bone density due to impaired osteoclastogenesis. In addition to osteoclasts, the overall mononuclear-phagocyte lineage development was compromised in these mice due to a reduced expression of the M-CSF receptor on myeloid progenitors. Although resting medullar hematopoiesis is normal in the absence of ERK1 [29], serial transplantation experiments revealed that ERK1 deficiency in the microenvironment alters the lodging and the functional activity of normal HSCs. Thus, our findings extend the role of ERK1 *in vivo*, through regulation of M-CSFR, as a key participant in the regulation of the niche size.

## Methods

### Mice

ERK1^−/−^ mice, backcrossed on C57BL/6 (CD45.2) background, and WT C57BL/6J (CD45.2 and CD45.1) mice (Charles River Laboratories,L'arbresle, France) were housed in a specific pathogen-free environment. All procedures were approved by the Animal Care Committee (registred no. P2.MG.137.10, 2010). Animal studies described herein were reviewed and approved (agreement no. 75-1064) by the Departmental director of veterinary services of Paris, France.

### Flow cytometry

For analysis of HSCs, BM cells were first stained with biotinylated-lineage cocktail antibodies (BD Biosciences) with additional biotinylated antibody specific for CD4, CD8, CD19 and NK1.1 (BioLegend), followed by streptavidin-PerCP secondary antibody (BioLegend) and PE-Cy7-conjugated anti-Sca1 (BioLegend), APC-eFluor780-conjugated anti-c-Kit (eBioscience), PE-conjugated anti-CD150 (BioLegend), Pacific Blue-conjugate anti-CD48 (BioLegend), APC-conjugated anti-CD135 (BD Pharmingen) and FITC-conjugated anti-CD34 (eBioscience). To analyze mature lineage cells, freshly isolated BM were immunostained with antibodies for myeloid cells (PE-conjugated anti-CD11b, FITC-conjugated anti-Gr1), B-cells (PE-conjugated anti-B220), T-cells (PE-conjugated anti-CD4, FITC-conjugated anti-CD8) (Biolegend). For analysis of BM monocytes and macrophages (MΦ), cells were preincubated with Fc blocking treatment, and subsequently stained with PE-conjugated anti-Gr1, APC-conjugated anti-CD115, and pacific blue-conjugated anti-F4/80 (MΦ). For phenotypic analysis of myeloid progenitors (CMP, GMP, MEP), cells were stained with the following biotinylated-lineage antibodies B220, CD11b, Gr1, Ter119, CD3ε and Il-7Rα (eBioscience, San Diego, CA). Lineage-positive cells were removed with BD IMag streptavidin particles (BD) and the remaining cells were stained with streptavidin-APC. Cells were incubated with PE/CY7-conjugated anti-Sca-1, PE-conjugated anti-c-Kit, APC-CY7-conjugated anti-FcγRII/III, and FITC-conjugated anti-CD34. The common myeloid progenitors (CMPs) were defined as Lin^−^Sca-1^−^IL7R^−^c-Kit^+^CD34^+^FcγRII/III^lo^, the granulo-macrophage progenitors (GMPs) cells as Lin^−^Sca-1^−^IL-7R^−^c-Kit^+^CD34^+^FcγRII/III^hi^, and the megakaryocyte-erythroid progenitors (MEPs) as Lin^−^Sca-1^−^IL-7R^−^c-Kit^+^CD34^−^FcγRII/III^lo^. All analyses were performed on a FACSCanto (BD Biosciences). Data were processed with the FlowJo software (TreeStar Inc). Myeloid progenitors (CMPs and GMPs) were sorted on the FACS Aria (BD Biosciences).

### Histomorphometric analysis

The right femur metaphysis was processed for histomorphometric analysis. The proximal halves of femur were fixed in 10% phosphate-buffered formaldehyde, dehydrated in methanol and embedded in methylmetacrylate resin without decalcification. Undecalcified 5-µm-thick longitudinal sections were prepared using a Leica 2055 microtome (Leica, Rueil-Malmaison, France) equipped with a tungsten carbide blade. Sections were stained with Goldner-Masson stain and histomorphometric indices were measured under blind conditions using a Leitz Aristoplan microscope (Leica) connected to a Sony DXC-930P color video camera. An automatic image analyzer (Microvisions Instruments, Evry, France) was used for bone parameter measurements. The trabecular bone volume (BV/TV), trabecular thickness (Tb.Th), trabecular number (Tb.N), trabecular separation (Tb.Sp) and osteoblast surface (ObS/BS) were measured in the secondary metaphyseal area of the proximal end of the femur in a standardized zone located at 300–500 µm from the growth cartilage and 150–200 µm from the cortices. The cortical thickness was measured at midshaft diaphysis. The left femurs were fixed in 4% paraformaldehyde, decalcified with 5% EDTA for 1 week and embedded in paraffin. Five-µm thick sections were dewaxed and processed for TRAP staining by incubation for 30 mn at 37°C in substrate solutions containing naphthol AS B1 phosphate (20 mg, Sigma), sodium tartrate (460 mg), fast red violet (6 mg) and N,N dimetyl formamide (1 Ml) in 20 ml acetate buffer (pH 5.0). Sections were then rinsed under running tap water for 5 min, counterstained with fast green dye and mounted in coverquick. The surface of TRAP-stained cells was measured for the Oc.S/BS calculation using automatic image analyzer (Microvisions Instruments, Evry, France).

### Homing and lodging assays

Total BM cells were isolated from donor animals (ERK1^−/−^ or WT). Cells were labeled with the cytoplasmic dye carboxyfluorescein diacetate succinimidyl diester (CFSE) according to manufacturer's instructions (Molecular Probes, Eugene, OR). In homing assays, lethally irradiated C57BL/6 mice were injected with 15×10^6^ ERK1^−/−^ or WT CFSE–stained cells. Three and 24 hours after injection, the percentage of CFSE^+^ cells in BM was scored by flow cytometry. In lodging assays, 15×10^6^ CFSE-labeled BM cells were transplanted into non irradiated mice. Three and 24 hours after injection, the percentage of CFSE^+^ cells in recipient-derived BM cells was scored by flow cytometry. The frequency of CFSE^+^ donor cells was determined as previously described [34].

### Limiting dilution competitive bone marrow transplantation (BMT) assay

For each BMT experiment, 50, 100 or 250 LSK CD150^+^CD48^−^ cells of each genotype were mixed with 25×10^4^ CD45.1/CD45.2 competitors, and subsequently transplanted into lethally (9,5 Gy) irradiated recipient mice CD45.1. Peripheral blood in recipient mice was analyzed for the engraftment level of donor cells at 4 and 20 weeks after BM transplantation. Peripheral blood was obtained by retro-orbital punction and blood leukocytes were obtained after hypotonic lysis. Multilineage hematopoietic engraftement was analyzed with FITC-conjugated CD45.1, APC-conjugated CD45.2 and the lineage markers Mac1 (CD11b), B220 and CD4 conjugated to (PE). All antibodies are from BioLegend. Data were analyzed with the FlowJo software (TreeStar Inc). Mice with over 1% donor descent-cells in the peripheral blood were counted as “positive reconstitution”. CRU frequencies were calculated using L-Calc software (StemCell Technologies). The data are pooled from 3 independent experiments.

### Serial transplantation in WT and ERK1^−/−^ mice

Lethally (9,5 Gy) irradiated 8- to 12-week old ERK1^−/−^ and WT CD45.2 mice were reconstituted with 2000 CD45.1 WT Lin^−^Sca-1^+^c-Kit^+^ (LSK) injected intravenously into the retro-orbital plexus in a total volume of 100 µL. Engraftment of primary recipient mice was analyzed after 20 weeks by flow cytometric analysis of peripheral blood. To test the self-renewal activity of the donor HSCs, bone marrow harvested from WT primary recipients after 20 weeks of engraftment was transplanted into lethally irradiated WT CD45.2 secondary recipients at a dose of 10^6^ cells per mouse. BM harvested from ERK1^−/−^ primary recipients was transplanted into lethally irradiated WT or ERK1^−/−^ CD45.2 secondary recipients. Flow cytometric peripheral blood analysis of the secondary recipients was performed 20 weeks after transplantation.

### *In vitro* osteoclastogenesis assays

Bone marrow from 8 week-old mice was flushed and mononuclear cells were purified by centrifugation over lymphocyte separation medium (Eurobio, France). Cells were seeded in 24-well plates at 10^5^ cells/well in 1 ml of alpha-MEM supplemented with 10% FBS, 25 ng/ml M-CSF, and 35 ng/ml recombinant RANKL (produced at SFR Biosciences UMS344/US8, PAP facility) [35]. The cultures were maintained for 5 days and re-fed every 2 days. Osteoclasts were identified by staining for TRAP activity, using the leukocyte acid phosphatase kit (Sigma Diagnostics, St. Louis, MO). TRAP-positive multinucleated cells with greater than three nuclei were counted as osteoclasts. RNA was isolated from the cells at day 5 of differentiation with cytokines to measure the expression of osteoclast-associated genes. To assess the osteoclast *in vitro* functionality, bone marrow-derived cells were differentiated *in vitro* for 4 days, and osteoclasts detached by treatment with 0,02% EDTA in PBS and numbered. For resorption test, the same number of osteoclasts from each set were lifted and seeded onto 24-well Osteo Assay dishes (Corning) for an additional 24 h. OsteoAssay wells were subsequently stained with silver nitrate [36]. The surface area was measured with ImageJ software, using an ImageJ plug-in developed by our imaging facility (Platim SFR Biosciences)

### Deoxypyridinoline cross-link assays

Bone related degradation products from type 1 collagen, deoxypyridinoline (DPD) cross-links and creatinin were measured in evening urine using the Pyrilinks-D immunoassay and creatinin kit (Quidel Corporation, San Diego, CA), according to the manufacturer's protocols.

### Monocyte/macrophage progenitors proliferation

L929-cell conditioned medium (LCCM) was used as a source of macrophage colony stimulating factor (M-CSF). Briefly, BM cells were separated by centrifugation over a Ficoll gradient and further purified by immunomagnetic depletion of CD45R- (B220), Ter119-, and CD3ε-positive cells. Enriched-mouse bone marrow monocytes were grown in DMEM-HG supplemented with 20% fetal calf serum, 30% LCCM, 100 U/ml penicillin, 100 µg/ml streptomycin, and 2 mM L-glutamine, and incubated at 37°C in a 5% CO_2_ atmosphere. Cell proliferation was evaluated at days 1, 3, and 7, using Uptiblue viable cell counting reagent (Interchim).

### *In vitro* phagocytic activity assay

Bone marrow-derived macrophages were cultured from WT and ERK1^−/−^ mice. Briefly, marrow was flushed from excised femur and cultured at 37°C in complete medium (DMEM supplemented with 20% FBS, 1% L-glutamine, 100 U/ml penicillin/streptomycin, and 30% LCCM). After 5 days of differentiation, the cells were scraped, and reseeded in 12-well plates at 2×10^5^cells/well. For latex bead uptake assay, green-yellow fluorescent latex beads (Molecular probes) were diluted 1∶300 with complete culture medium and incubated for 30 minutes at 37°C. Non-adherent latex beads were removed by vigorous washing. Cells were then harvested by treatment with 5 mM EDTA and scraping. The amount of phagocytozed beads was measured by flow cytometry on F4/80+-gated population. The amount of nonspecifically bound beads was determined by incubating macrophages with fluorescent beads on ice. The experiment was done with macrophages cultured from 3 mice for each genotype.

### RNA extraction and quantitative real-time PCR (qPCR)

Total RNA was isolated from the various samples using RNeasy mini kit with on-column genomic DNA digestion on column step according to the manufacturer's instructions (Qiagen, Valencia, CA). RNA was then reverse transcribed with random hexamer primers using Superscript II (Invitrogen). qPCR was performed with SYBR Green PCR Master mix on a lightcycler (Roche Diagnostics, Mannheim, Germany). Sequences of primer pairs used in these qPCR experiments are listed in Table S1. Data were analyzed using the ΔΔ–Ct relative quantification with each duplicate sample normalized to actin.

### Statistical analysis

All experimental data were analyzed and compared for statistically significant differences by two-tailed Student's *t* test or the non parametric Mann-Whitney test, as indicated. Data are presented as the averaged values ± standard error of the mean (SEM). All *P* values reported are of comparisons made to results obtained with WT animals. The following values were considered significant: *, *P*<0.05, **, *P*<0.01, and ***, *P*<0.001.

## Results

### ERK1 deletion increases bone density *in vivo*

**Figure 1:**
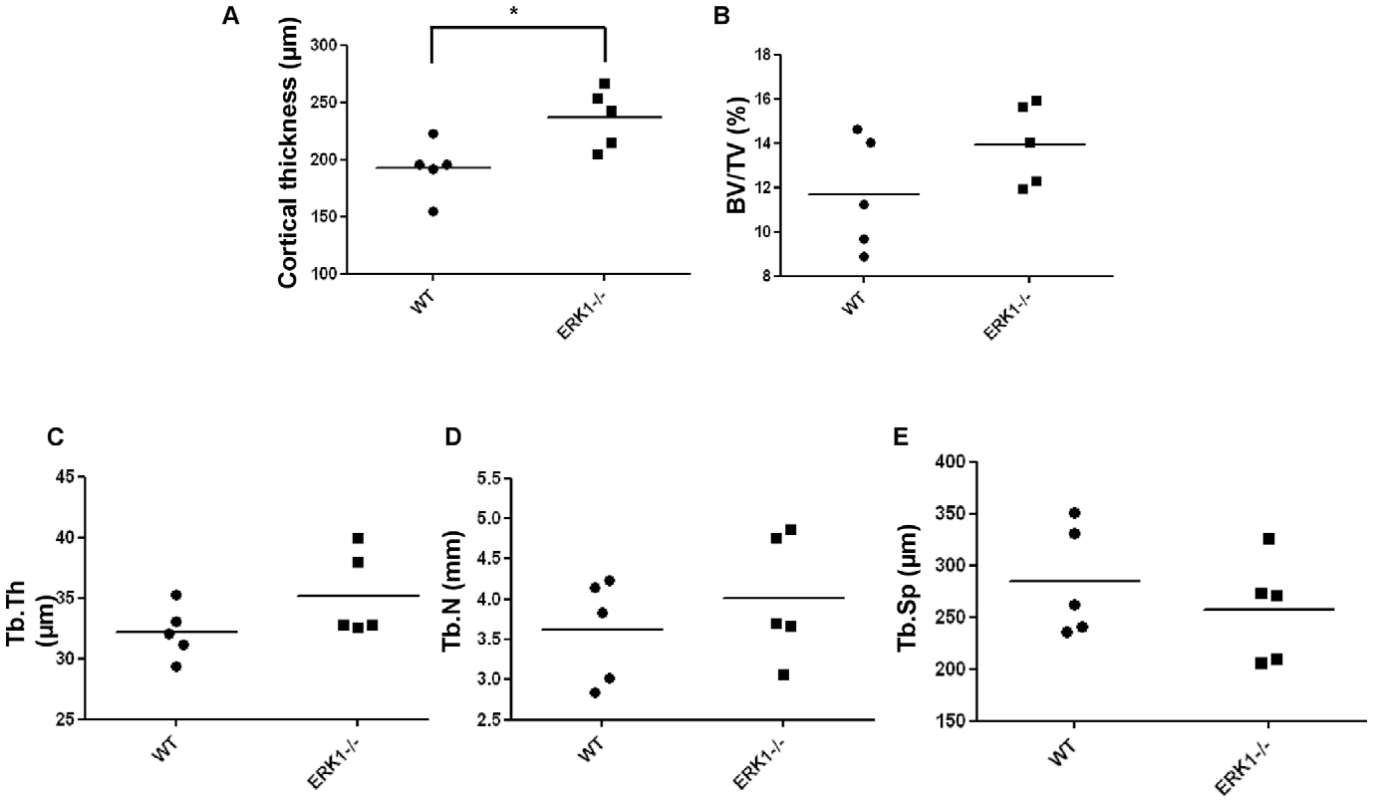
ERK1 loss alters the bone architecture. Cortical and trabecular parameters in WT and ERK1^−/−^ mice (n = 5 for each group). (A) midshaft diaphysis cortical thickness, (B) bone volume/tissue volume (% BV/TV), (C) trabecular thickness (Tb.Th), (D) trabecular number (Tb.N), (E) trabecular separation (Tb.S). The non parametric Mann-Whiney test was used to calculate the *P* value. Horizontal bars show the mean values.

Abnormal bone formation was observed in the ERK1^−/−^ mice. Histomorphometric measurements of WT and ERK1^−/−^ mice femurs were applied to analyze the trabecular and cortical microarchitecture. Data indicated that the cortical bone mass of ERK1^−/−^ mice showed a significant increase (Figure 1A). The trabecular bone volume/tissue volume (BV/TV) increased in ERK1^−/−^ mice, though the significance was not reached (Figure 1B). Likewise not statistically significant, the analysis of the main trabecular parameters, including trabecular thickness (Tb.Th, Figure 1C), trabecular number (Tb.N, Figure 1D), and trabecular separation (Tb.Sp, Figure 1E) showed differences between WT and KO animals in accordance to the increased BV/TV. Though significance was not reached, this finding suggests that bone-remodeling processes are impaired in ERK1^−/−^ mice.

### Bone marrow lodging and homing are altered in the absence of ERK1

**Figure 2:**
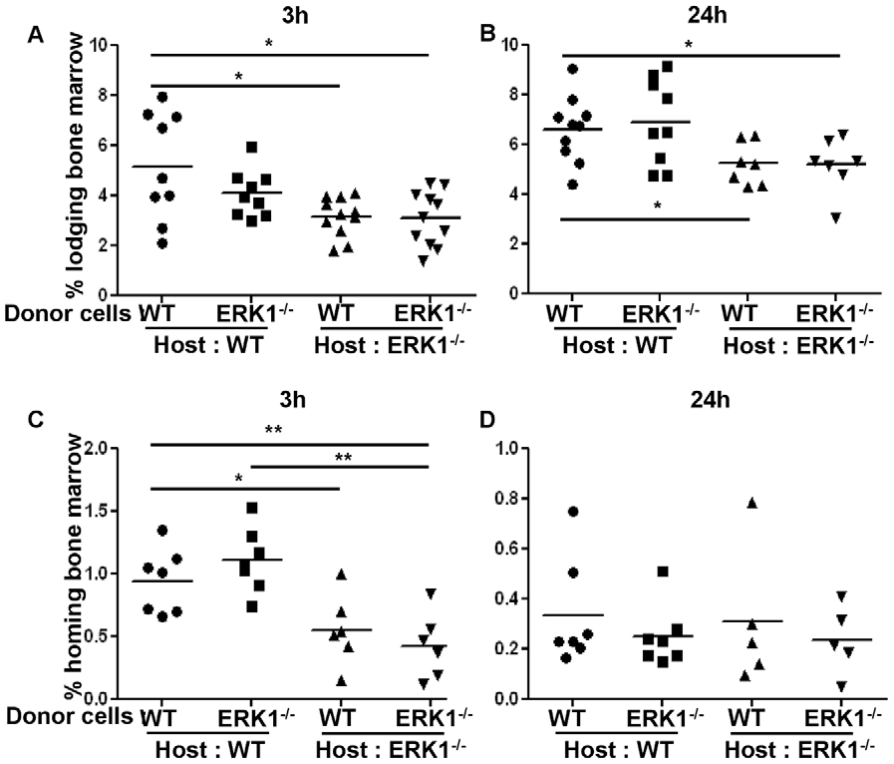
ERK1^−/−^ microenvironment alters the lodging and the homing efficiencies of BM cells. Lodging of the bone marrow cells into a non irradiated host were quantified 3 h (A) and 24 h (B) after injection. Homing of the bone marrow cells into a lethally irradiated host were quantified 3 h (C) and 24 h (D) after injection. Results are presented as scatter plots showing the percentage of recovered CFSE^+^ cells in the BM 3 and 24 hours after transplantation. The non parametric Mann-Whiney test was used to calculate the *P* value. Horizontal bars show the mean values.

An abnormal bone formation may have a major impact on the BM microenvironment and, consequently on the lodging and homing abilities of HSCs. In order to study these properties, WT and ERK1^−/−^ BM cells were labelled with CFSE before being transplanted into lethally irradiated (homing) or not irradiated (lodging) WT or ERK1^−/−^ recipients. 15×10^6^ BM labelled-cells were injected in non-irradiated recipient mice. WT and ERK1^−/−^ BM cells were found to lodge similarly in WT recipient mice at 3 hours (Figure 2A) or 24 hours (Figure 2B). However WT and ERK1^−/−^ BM cells both presented a striking defect in their ability to lodge in ERK1^−/−^ recipient mice after 3 and 24 hours (Figure 2A and B, respectively). Similarly, a defect in homing of WT and ERK1^−/−^ BM cells in ERK1^−/−^ mice at 3 hours was also observed (Figure 2C) which was not longer seen after 24 hours (Figure 2D). These results indicate that the lodging and the homing abilities of the ERK1^−/−^ HSC are impaired.

### The hematopoietic bone marrow microenvironment is defective in the absence of ERK1

**Figure 3:**
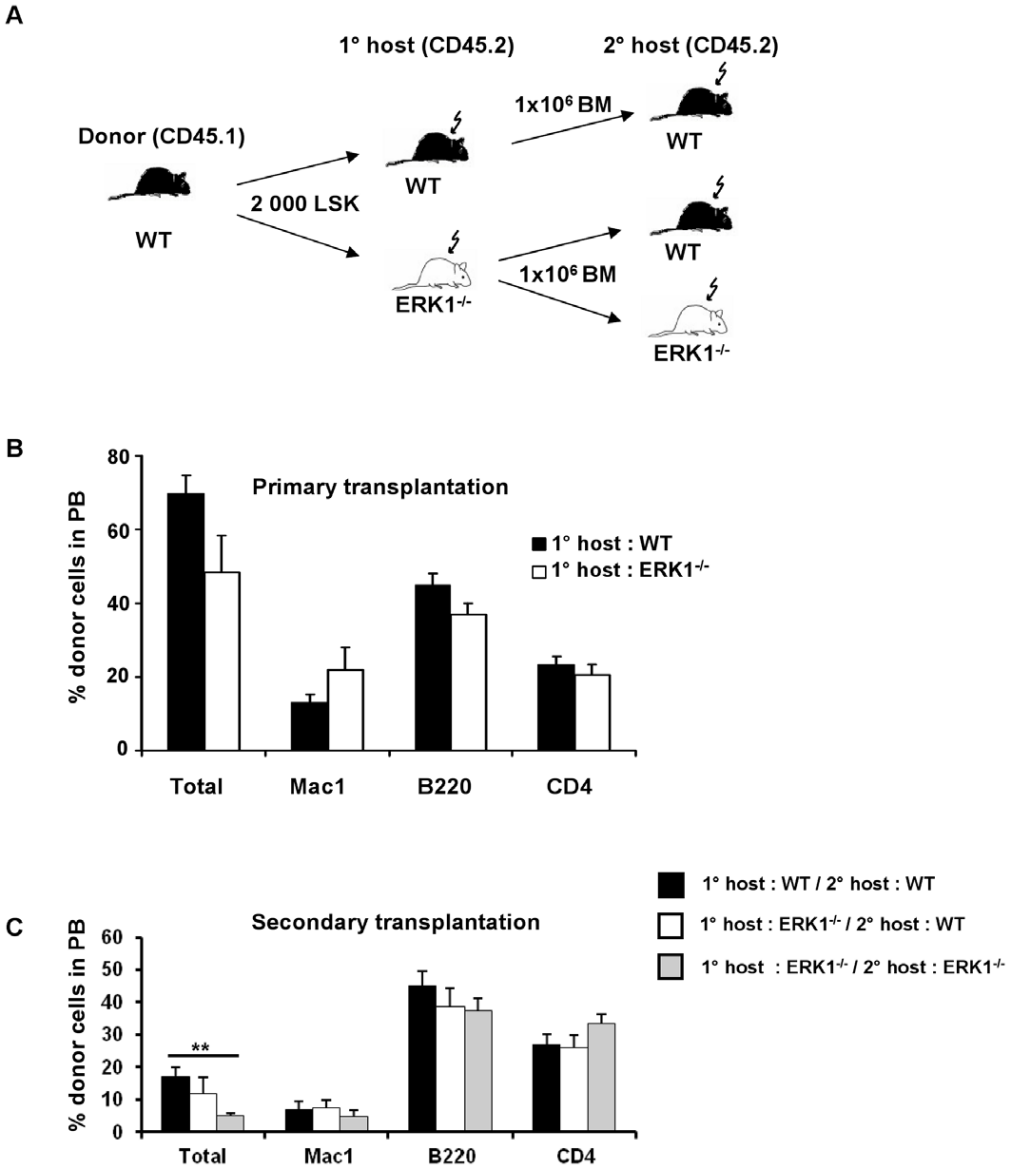
ERK1^−/−^ microenvironment induces a defect in WT HSC activity. (A) Scheme of transplantation assay. (B) Analysis of WT donor cells engraftment in WT (n = 10) and ERK1^−/−^ mice (n = 10) primary recipient 24 weeks after transplantation. (C) Analysis of WT donor cells engraftment in WT (n = 9 per group) and ERK1^−/−^ mice (n = 16) secondary recipient 24 weeks after transplantation. In panels B and C, total donor cells are shown as a percentage of live cells. Individual lineages are shown as a percentage of donor-derived cells. Data represent the mean±SEM, the *t*-test was used to calculate the *P* value.

The ERK1^−/−^-defective environment may affect the engrafment of HSCs in competitive repopulation assays. Lethally irradiated 8- to 12 week-old ERK1 mutant and littermate control mice (both CD45.2 genotype) were transplanted with 2 000 Lin^−^Sca1^+^c-kit^+^ (LSK) isolated from wild-type CD45.1 mice (Figure 3A). Long term and multilineage engraftment of primary recipients was confirmed by flow cytometry analysis of peripheral blood (Figure 3B). WT donor cells that had been transplanted in ERK1^−/−^ recipients reconstituted hematopoiesis to a lesser extent, even though this was not statistically significant (Figure 3B). In order to test the self-renewal activity of the donor HSCs, BM harvested from WT and ERK1^−/−^ primary recipients after four months of engraftment was transplanted into lethally irradiated WT or ERK1^−/−^ secondary recipients at a dose of 10^6^ cells per mouse (Figure 3A). Flow cytometric analysis of PB of the secondary recipients 4 months after transplantation revealed that WT donor cells passaged through a primary mutant microenvironment reconstituted hematopoiesis to a much lower extent when transplanted in a ERK1^−/−^ second recipient (2.9-fold decreased, *P* = 0.0005), compared with WT cells passaged through a primary mutant microenvironment and then transplantated in a secondary WT recipient (Figure 3C). These data strongly support the hypothesis that ERK1 is involved in the maintenance of an appropriate medullar microenvironment, but that the intrinsic properties of HSCs are not altered by the ERK1^−/−^ defective microenvironment. In agreement with that, the frequency of functional ERK1^−/−^ HSCs (CRU) is identical to that of WT HSCs when tested in limiting dilution conditions (Table S2).

#### Development and maturation of osteoclasts are impaired in the absence of ERK1

**Figure 4:**
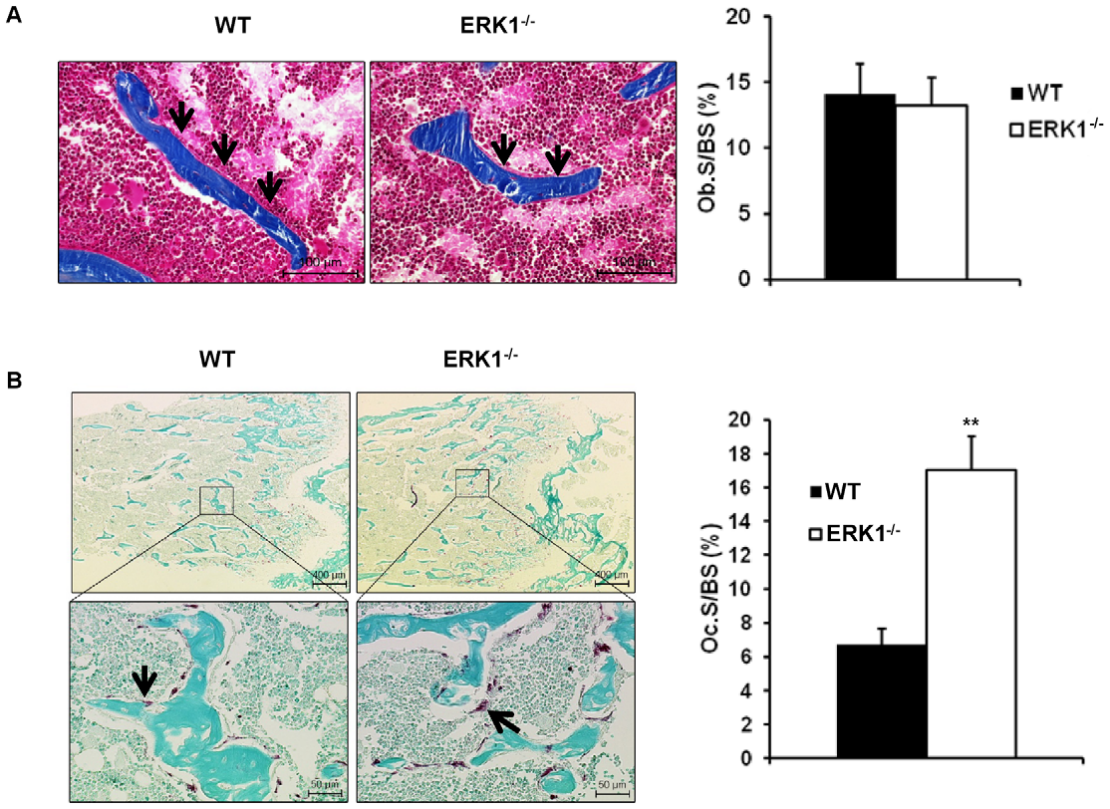
Deletion of ERK1 impairs osteoclast formation without any effects on osteoblasts. WT and ERK1^−/−^ mouse femur sections were stained with Trichrome Goldner-Masson stain (A) and revealed for TRAP activity (B). Ob.S/BS and Oc.S/BS parameters were calculated from Goldner trichrome and TRAP activity sections, respectively. Data are the means±S.E.M. (n  = 5 per group). Data represent the mean±SEM, the Mann-Whitney test was used to calculate the *P* value.

**Figure 5:**
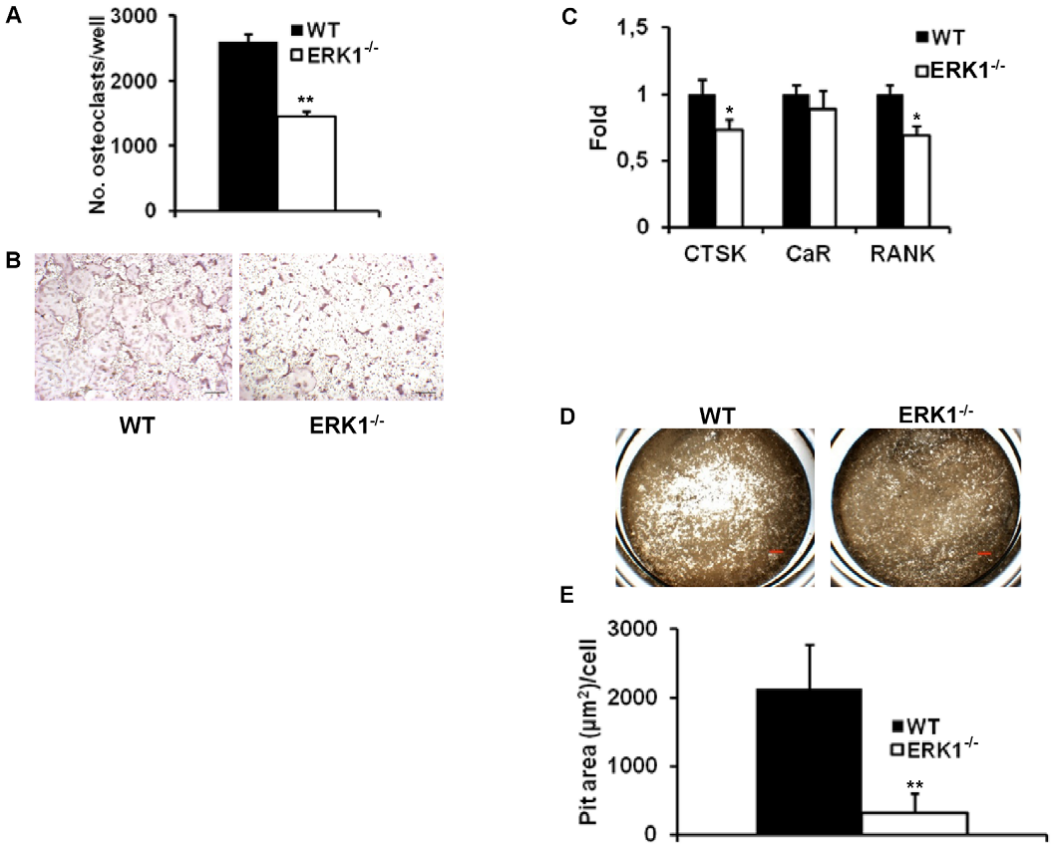
ERK1 deficiency impairs osteoclastogenesis. (A) Total number of osteoclasts per well following 5 days of culture under osteoclastogenic condition (n = 3) (B) Representative picture of TRAP-positive multinucleated osteoclasts generated from BMNCs of WT and ERK1^−/−^ mice. (C) Relative expression of cathepsin K (CTSK), calcitonin receptor (CaR), and receptor activator of nuclear factor *kappa* B (RANK) in WT and ERK1^−/−^ osteoclasts. (D) Representative pictures of the bone resorption pits formed by WT and ERK1^−/−^ derived osteoclasts. (E) Bar graph representing the quantification of the resorptive area per dentin slice for WT and ERK1^−/−^ osteoclasts (n = 3). Data represent the mean± SEM. The *t*-test was used to calculate the *P* value.

Among the cells involved in these specialized microenvironments, osteoclasts and osteoblasts play major roles. Bone homeostasis is maintained by a tight regulation between bone-forming osteoblasts (OBs), and bone-resorbing osteoclasts (OCs). To address whether the bone phenotype observed in ERK1^−/−^ mice is a consequence of a deregulation of the balance between OBs and OCs, these cells were quantified on bone sections. No difference in OB number between WT and ERK1^−/−^ animals was observed (Figure 4A). Furthermore, the expression of osteoblast-associated genes was similar between the two genotypes (Figure S1). These data suggest that OBs were not altered in ERK1^−/−^ mice. We next determined if increased bone in ERK1^−/−^ mice is a consequence of impaired bone resorption. TRAP staining of bone sections demonstrated a significant increase in the numbers of ERK1^−/−^ TRAP+ cells along the trabecular endosteum, in comparison to their WT counterparts (Figure 4B). To evaluate if ERK1 deletion leads to impaired OC function, we tested the differentiation potential of ERK1^−/−^ and WT BMMCs cultured with M-CSF and RANKL. Following 5 days of differentiation, TRAP staining of WT and ERK1^−/−^ osteoclasts grown *in vitro* revealed a dramatic impairment of osteoclastogenesis in KO cultures with a reduction of osteoclast number by almost 2 fold (Figure 5A). In addition, these ERK1^−/−^ osteoclasts appeared smaller, with a reduced ability to form multinucleated cells (Figure 5B). Transcriptional assessment of the osteoclast-associated genes RANK and cathepsin K (CTSK), after 5 days in culture under osteoclastogenic conditions, revealed that ERK1^−/−^ BM cells exhibited significant reduced expression of these markers of terminal osteoclastic differentiation, while the mRNA levels of the calcitonin receptor (CaR) showed only a modest decrease (Figure 5C). Osteoclast activity was further assessed by analyzing dentin resorption pits after 6 days of culture. Quantification of the resorption areas, normalized to the numbers of osteoclasts, revealed a drastic reduction (∼85%) in the resorptive capacity of ERK1^−/−^ osteoclasts as compared to their wild-type counterparts (Figures 5D–E). To address the functionality of OCs *in vivo*, we measured the urinary deoxypyridinoline (DPD) concentration. DPD is the product of the degradation of type I collagen and is a read-out of the ongoing bone resorption process. Urinary excretion of DPD was lower in ERK1^−/−^ mice compared to WT (6.72±0.27 vs 5.52±0.58 nmol/mmol creatinine, *P* = 0.06, n = 8 for each genotype). These results suggest that ERK1 is required for the regulation of normal osteoclast development.

### ERK1 loss alters bone marrow monocyte frequency *in vivo* and monocytes progenitors proliferation *in vitro*

**Figure 6:**
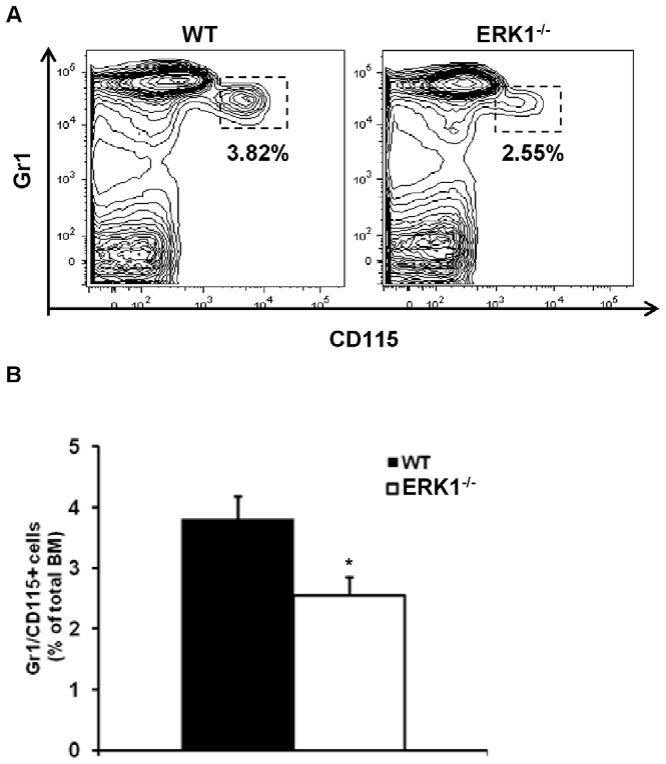
ERK1^−/−^ mice have a reduced fraction of bone marrow monocytes. (A) Representative FACS plot of BM monocytes defined as Gr1^+^CD115^+^ cells. (B) Proportion of monocytes as described above in the total BM in WT (n = 11) and ERK1^−/−^ (n = 12). Data represent the mean±SEM. The *t*-test was used to calculate the *P* value.

As osteoclasts derive from precursors of the monocyte/macrophage lineage, investigation of the monocytic compartment was performed to fully characterize the ERK1 cellular target. Monocytes originate in the bone marrow in a CD115 (M-CSFR)-dependent manner from a myeloid progenitor [37]. We therefore used Gr1 and CD115 antibodies to identify BM monocytes by flow cytometry [38]. Results showed a significant reduction of the frequency of Gr1^+^/CD115^+^ monocytes in ERK1^−/−^ mice (Figure 6A, B). Furthermore, the mean fluorescence intensity (MFI) of CD115 was also reduced, indicating that the overall expression of the receptor in this population is decreased (3674±317 for WT vs 3244±234 for ERK1^−/−^). We next investigated if the reduced number of monocytes in the BM may be a consequence of an altered proliferation of monocyte progenitors. BM mononuclear-enriched myeloid cells isolated from WT and ERK1^−/−^ mice were grown in L929-conditioned medium for up to 7 days and tested for their proliferation ability. The results obtained showed that deletion of ERK1 delayed M-CSF-induced monocytes progenitors proliferation (Figure S2). These overall data suggest that ERK1 contribute to the regulation of monocytes progenitors proliferation through the activation of the M-CSF signaling.

### ERK1 deletion does not affect macrophage differentiation and functionality

We further investigated if the alteration of the response to M-CSF *in vitro* was correlated with a decrease of mature macrophages number *in vivo*. The splenic macrophages have been characterized as F4/80^hi^CD11b^lo/−^ cells with strong autofluorescence [39], [40]. The WT and the ERK1^−/−^ spleen cells were stained with antibodies to F4/80 and CD11b.The results obtained showed no difference between the ERK1^−/−^ and WT mice (1.18%±0.09% for WT and 1.25%±0.21% for ERK1^−/−^,*P* = 0.36, n = 6 for both groups). Recently, bone marrow resident macrophages have been described based on differential expression of Gr1, CD115, and F4/80 [38]. According to this labeling, BM macrophages were identified in WT and ERK1^−/−^ mice, and showed no significant difference in their frequency (Figure S3). These data suggest that *in vivo*, mechanisms can compensate for the reduction of monocyte/macrophage progenitors, resulting in a normal distribution of macrophages in the organs under steady-state conditions. In particular, granulocyte-macrophage CSF (GM-CSF) and IL-3 contribute to M-CSF-independent recovery for some macrophages populations in M-CSF-deficient mice [19]. We further investigated the functionality of macrophages from WT and ERK1^−/−^ mice. The phagocytic activity of BM-derived macrophages (BMM) was evaluated by measuring the capacity of the cells to ingest latex beads *in vitro*. The results showed that phagocytosis efficiency was similar in WT and ERK1^−/−^ BMM (Figure S4).

### ERK1 deletion alters the myeloid progenitors compartment

**Figure 7:**
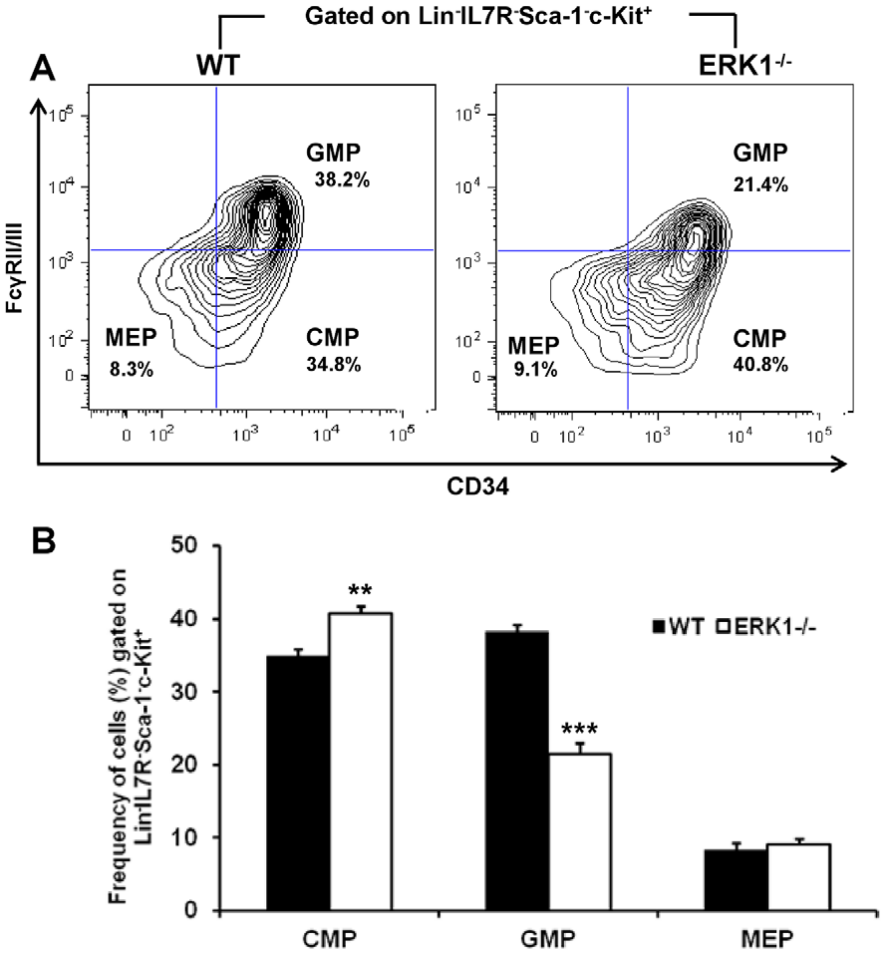
ERK1 deficiency alters the myeloid lineage differentiation. (A) Representative FACS plot of CMP, GMP, and MEP in WT and ERK1^−/−^ mice. (B) Frequency of CMP, GMP, and MEP populations in WT and ERK1^−/−^ mice calculated according to the gating strategy shown in panel A. Values are derived from 6 mice of each genotype. Data represent the mean±SEM. The *t*-test was used to calculate the *P* value.

*In vivo* analysis of the myeloid lineage progenitors, CMP, GMP and MEP subsets revealed a significant phenotypical change in the CMP and GMP subsets in the ERK1^−/−^ mice (Figure 7A). The frequency of CMPs increased by approximately 1.3-fold, while the frequency of GMPs significantly decreased by almost 2-fold, compared with the respective WT compartments (Figure 7A, B). In parallel, the frequency of MEPs showed no change between WT and ERK1^−/−^ mice. These data suggest that ERK1 deletion alters the differentiation of CMPs into GMPs, while it does not affect the MEP commitment and that the CMPs are the cellular targets of ERK1. We have previously shown that ERK1 loss impairs both the osteoclasts and the bone marrow monocytes. As these cells originate in the bone marrow in a CD115 (M-CSFR)-dependent manner from a myeloid progenitor, we next quantified the expression of CD115 in the GMP and CMP subsets. We observed a 2-fold reduction of the GMPs expressing CD115 (14.1%±3% and 6.96%±2.8% for WT and ERK1^−/−^ mice, respectively, *P* = 0.04; n = 3). Furthermore, the mean fluorescence intensity of CD115 was lower in ERK1^−/−^ GMPs compared to WT (MFI: 2161±190 vs 2550±119; *P* = 0.04; n = 3). When gated on the CMP subset, the fraction of CD115-positive cells was too low for interpretation. To get insight in the regulation of CD115 expression in myeloid progenitors, we next measured the expression of CD115 mRNA in WT and ERK1^−/−^ GMP-sorted populations. The mRNA expression of CD115 was found to be similar in WT and ERK1^−/−^. No difference in the level of CD115 mRNA was found between WT and ERK1^−/−^ GMP nor CMP, though the level of CD115 gene expression is very low in these latter cells (Figure S5). These results show that the cellular targets of ERK1 are M-CSFR-responsive cells, upstream to osteoclasts.

## Discussion

In this study we aimed to decipher the role of ERK1 *in vivo* in the regulation of HSCs. We show that ERK1^−/−^ mice exhibit an overall increase in bone density. These skeletal abnormalities are caused by a deficiency of the osteoclast function. In agreement with these data, mice deficient for ERK1 develop a mild osteopetrosis. Our results provide evidence that ERK1 is involved in the maintenance of an appropriate medullar microenvironment and plays a role in the cells that comprise the hematopoietic microenvironment.

Our results indicate that ERK1 deletion causes defective lodging and homing. This defect results in impaired capacity of WT cells to reconstitute when serially transplanted in ERK1^−/−^ mice. This lower capacity was more pronounced when WT donor cells passaged through a primary mutant microenvironment were transplanted in a ERK1^−/−^ second recipient mice. This result reveals that the negative impact on hematopoietic recovery is strictly dependent of the mutant BM microenvironment. By contrast, the deletion of ERK1 was without effect on the functional properties of HSCs. An altered BM niche could explain the defective effects observed. Specifically, ERK1 may be necessary in cell types implicated in the regulation of the niche size. Previous studies have introduced the link between bone remodeling and the regulation of hematopoiesis, suggesting a dynamic nature of the BM stem cell niche [7]. Among the cellular components of the medullar niches, osteoclasts are have been shown to be involved in the hematopoietic niche homeostasis [41], [42], controlling both the maintenance and the release of HSC. In the ERK1^−/−^ mice, the major default of *in vitro* osteoclastogenesis contrasts with the *in vivo* increased number of osteoclasts. This could be due to an increased production of osteoclasts to compensate their functional defect. Indeed, similar processes of regulation have been previously described in other osteoclast-defective models [43], [44], [45]. While this paper was in preparation, Y. He et al. also reported a major defect of bone resorbing activity in ERK1^−/−^ mice [33]. However, they showed that in addition to both *in vitro* and *in vivo* bone-resorbing activity alterations, the number of osteoclasts was markedly reduced in ERK1^−/−^ trabecular area. Variations between these results and ours may reflect differences in the conditions of animal housing and/or ERK1^−/−^ mouse line maintenance, resulting in phenotypes with various degrees of severity. However, although the osteopetrotic phenotype described by He and colleagues was more severe than that we observed, the cellular mechanisms underlying this default are the same.

In addition to the defect in osteoclastogenesis, we report an overall compromised mononuclear-phagocyte lineage development in ERK1^−/−^ mice, through the alteration of M-CSFR expression. Mice defective for M-CSFR (*Csf1r^−^/Csf1r^−^*)mice are severely osteopetrotic, with reduced mononuclear phagocyte production, reduced number of macrophages, and development of a splenomegaly [9]. We found similar characteristics in our model, but with a less severe phenotype. This corroborates the reduced expression of M-CSFR^+^ cells in ERK1^−/−^ mice. It is well-known that ERK is a downstream effector of M-CSFR and that it contributes to the regulation of myelopoiesis. However, it is to our knowledge, the first demonstration of a control of M-CSFR expression downstream of ERK1. The loss of ERK1 leads to impaired expression of the M-CSFR in myeloid progenitors and induce a delay in their differentiation program, as shown by the accumulation of CMPs in ERK1^−/−^ bone marrow, while the proportion of GMPs was reduced. The fraction of CD115-positive cells on the CMP subset was too low for interpretation. The CMPs have been shown to be divided into three phenotypically, functionally and developmentally distinct cell subsets [46] that express or not the M-CSFR. In our study we quantified the M-CSFR expression on the overall CMP population. The M-CSFR expression study in the three described subsets in the ERK1^−/−^ mice may allow a quantification of the M-CSFR and explain the increase of the CMP compartment. Furthermore, we reported a decrease in the BM monocytic population (Gr1^+^/CD115^+^) in ERK1^−/−^ mice. Together, these data suggest that ERK1 plays a key role in myelopoiesis by controlling the expression of M-CSFR. This control does not take place at the transcriptional level, as the expression of the M-CSFR mRNA is not different between WT and ERK1^−/−^, in neither GMP or CMP myeloid progenitors. This means that receptor synthesis is more likely to be governed by posttranscriptional regulation as previously suggested [47]. Thus, we can hypothesize that ERK1 is involved in the posttranscriptional mechanisms regulating the expression of the M-CSFR. In line with this hypothesis, it has been shown that the ERK pathway is involved in the mechanisms that control M-CSFR turnover [48].

In summary, our results show that the microenvironmental default observed in ERK1^−/−^ mice is in fact a consequence of an erroneous myeloid lineage commitment. It has been recently published that the inhibitor of differentiation (Id1) gene plays a role in regulation of bone and BM physiology [20]. In Id1^−/−^ mice, the osteoporosis phenotype and increased osteoclasts observed are suggested to be due to a decrease of the CMP population. Likewise, our study suggests that GMPs/CMPs are the ERK1 potential cellular targets. The ERK1-regulated M-CSFR expression characterizes one of the mechanisms involved. While ERK1 is well known to be activated by M-CSF, the present results are the first to point out an ERK1-dependent M-CSFR regulation on hematopoietic progenitors. The observed bone and BM microenvironment phenotypes as well as the decreased osteoclastogenesis in ERK1^−/−^ mice are due to an altered homeostasis of the myeloid precursors cells. This study reinforces the hypothesis of an active cross-talk between HSCs, their progeny and bone cells in the maintenance of the homeostasis of these compartments.

## Supporting Information

Figure S1 **qPCR analysis of the osteoblast-associated genes RUNX2, alkaline phosphatase (ALP), osteocalcin (OSC), osteopontin (OPN) in WT and ERK1^−/−^ bone samples.** Data are presented as the mean ± SEM, n = 3 for each genotype.
(TIF)

Figure S2 **M-CSF induced proliferation of BM monocyte/macrophage progenitors.** Enriched monocyte/macrophage progenitors were grown *in vitro* in L929-conditioned medium for up to 7 days. Cell proliferation was monitored by means of the fluorimetric metabolic growth indicator Uptiblue (Interchim) at the indicated days. Data show that deletion of ERK1 induces a delay in growth of cells during the first days of culture.
(TIF)

Figure S3 **Gating strategy for the BM macrophages.** Gated on the Gr1^−^ population, F4/80^+^CD115^−^ cells were further subdivided on a FSC/SSC plot. SSC^int/lo^ cells are considered as macrophages. Results are representative of 3 independent experiments, for a total of 9 mice for each genotype.
(TIF)

Figure S4 **Bead incorporation of BM-derived macrophages.** BM-derived macrophages were incubated for 30 minutes with fluorescent latex beads at 37°C. Cells were then detached, and the fluorescence distribution was evaluated by FACS on F4/80^+^ gated population. No difference was seen between WT and ERK1^−/−^ macrophages. The histograms represent the percentage of macrophages that incorporated the indicated number of beads (n = 3 mice for each genotype).
(TIF)

Figure S5 **qPCR analysis of M-CSFR gene expression in WT and ERK1^−/−^ CMPs and GMPs.** Data are presented as the mean ± SEM, n = 5 for each genotype.
(TIF)

Table S1 **Primer sequence list.**
(TIF)

Table S2 **CRU frequencies of WT and ERK1^−/−^ HSCs.**
(TIF)
