# Supplementary figures and images for: ERK1 Regulates the Hematopoietic Stem Cell Niches
Source: PLoS One. 2012 Jan 30;7(1):e30788. doi: 10.1371/journal.pone.0030788 (PMC3268766; doi:10.1371/journal.pone.0030788)

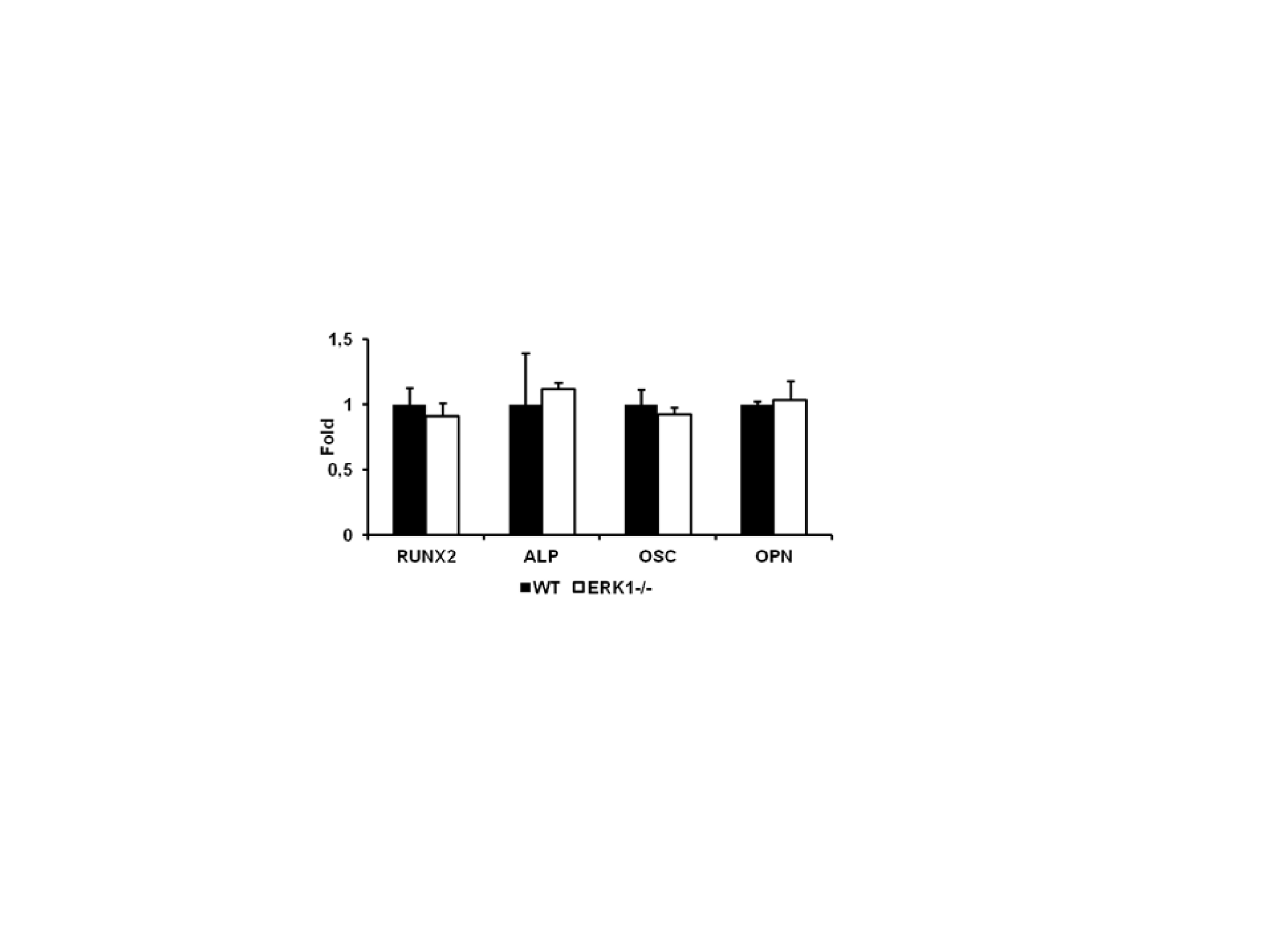

Supplement: Figure S1 — qPCR analysis of the osteoblast-associated genes RUNX2, alkaline phosphatase (ALP), osteocalcin (OSC), osteopontin (OPN) in WT and ERK1−/− bone samples. Data are presented as the mean ± SEM, n = 3 for each genotype. (TIF) [file pone.0030788.s001.tif]

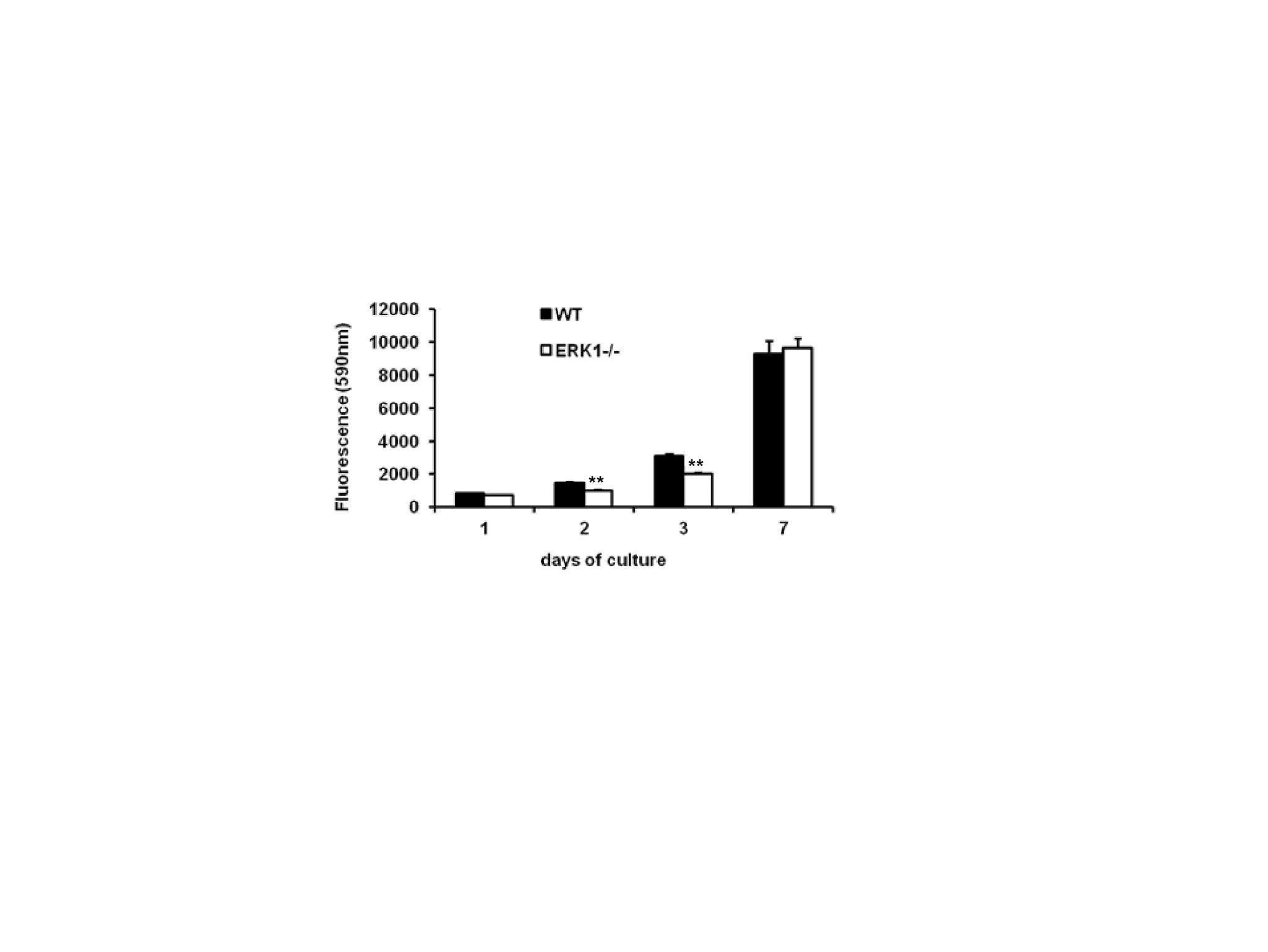

Supplement: Figure S2 — M-CSF induced proliferation of BM monocyte/macrophage progenitors. Enriched monocyte/macrophage progenitors were grown in vitro in L929-conditioned medium for up to 7 days. Cell proliferation was monitored by means of the fluorimetric metabolic growth indicator Uptiblue (Interchim) at the indicated days. Data show that deletion of ERK1 induces a delay in growth of cells during the first days of culture. (TIF) [file pone.0030788.s002.tif]

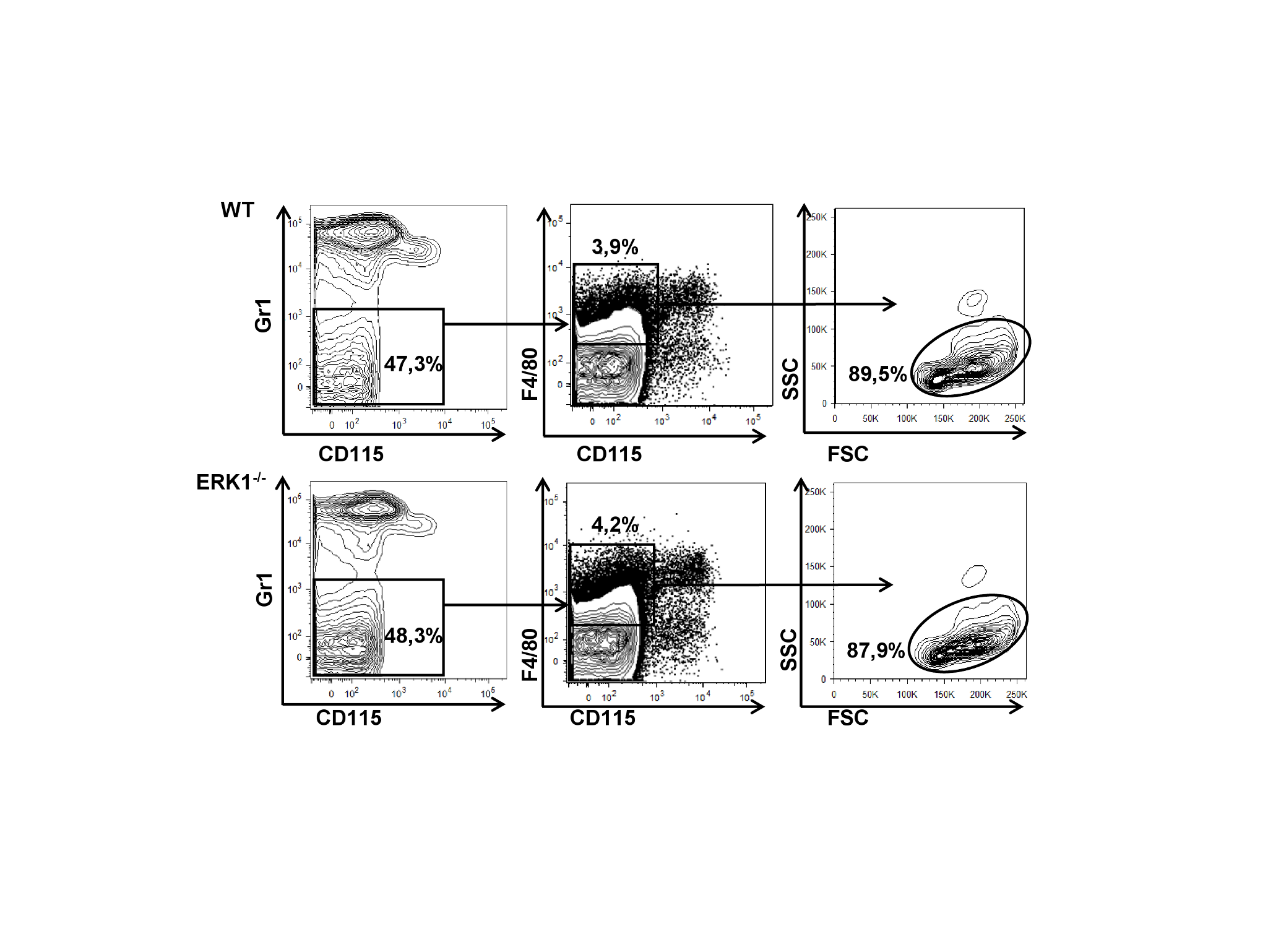

Supplement: Figure S3 — Gating strategy for the BM macrophages. Gated on the Gr1− population, F4/80+CD115− cells were further subdivided on a FSC/SSC plot. SSCint/lo cells are considered as macrophages. Results are representative of 3 independent experiments, for a total of 9 mice for each genotype. (TIF) [file pone.0030788.s003.tif]

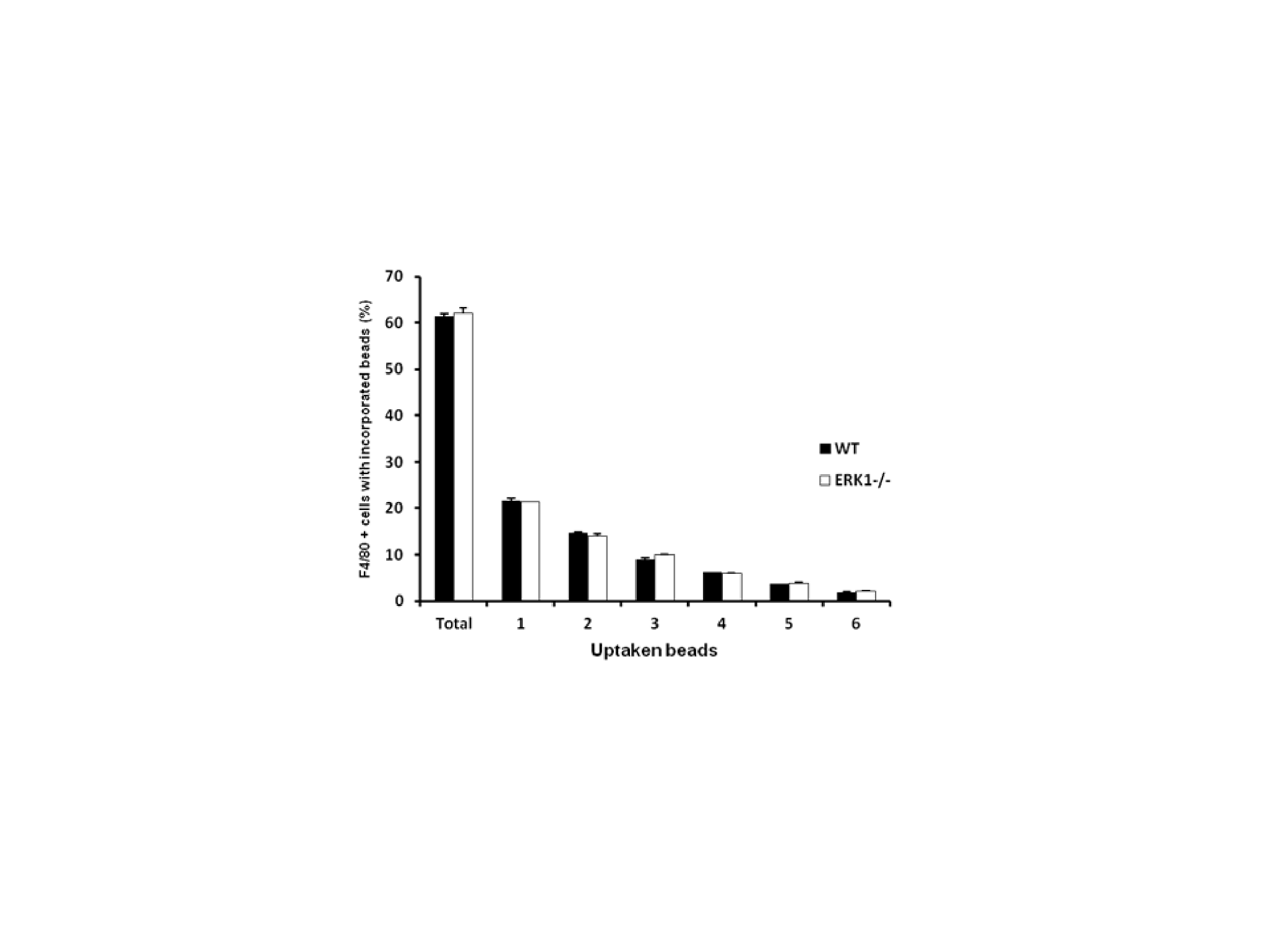

Supplement: Figure S4 — Bead incorporation of BM-derived macrophages. BM-derived macrophages were incubated for 30 minutes with fluorescent latex beads at 37°C. Cells were then detached, and the fluorescence distribution was evaluated by FACS on F4/80+ gated population. No difference was seen between WT and ERK1−/− macrophages. The histograms represent the percentage of macrophages that incorporated the indicated number of beads (n = 3 mice for each genotype). (TIF) [file pone.0030788.s004.tif]

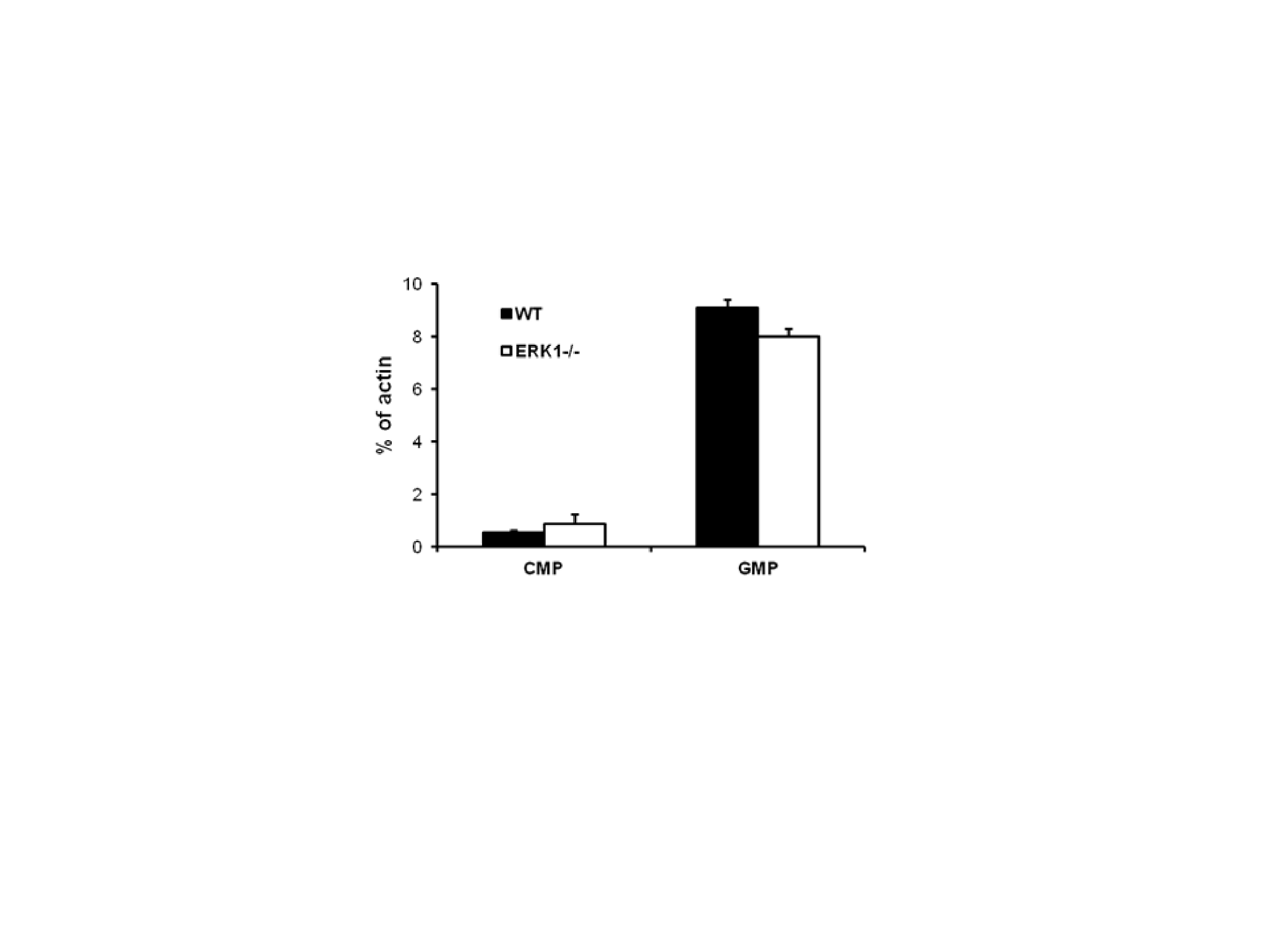

Supplement: Figure S5 — qPCR analysis of M-CSFR gene expression in WT and ERK1−/− CMPs and GMPs. Data are presented as the mean ± SEM, n = 5 for each genotype. (TIF) [file pone.0030788.s005.tif]

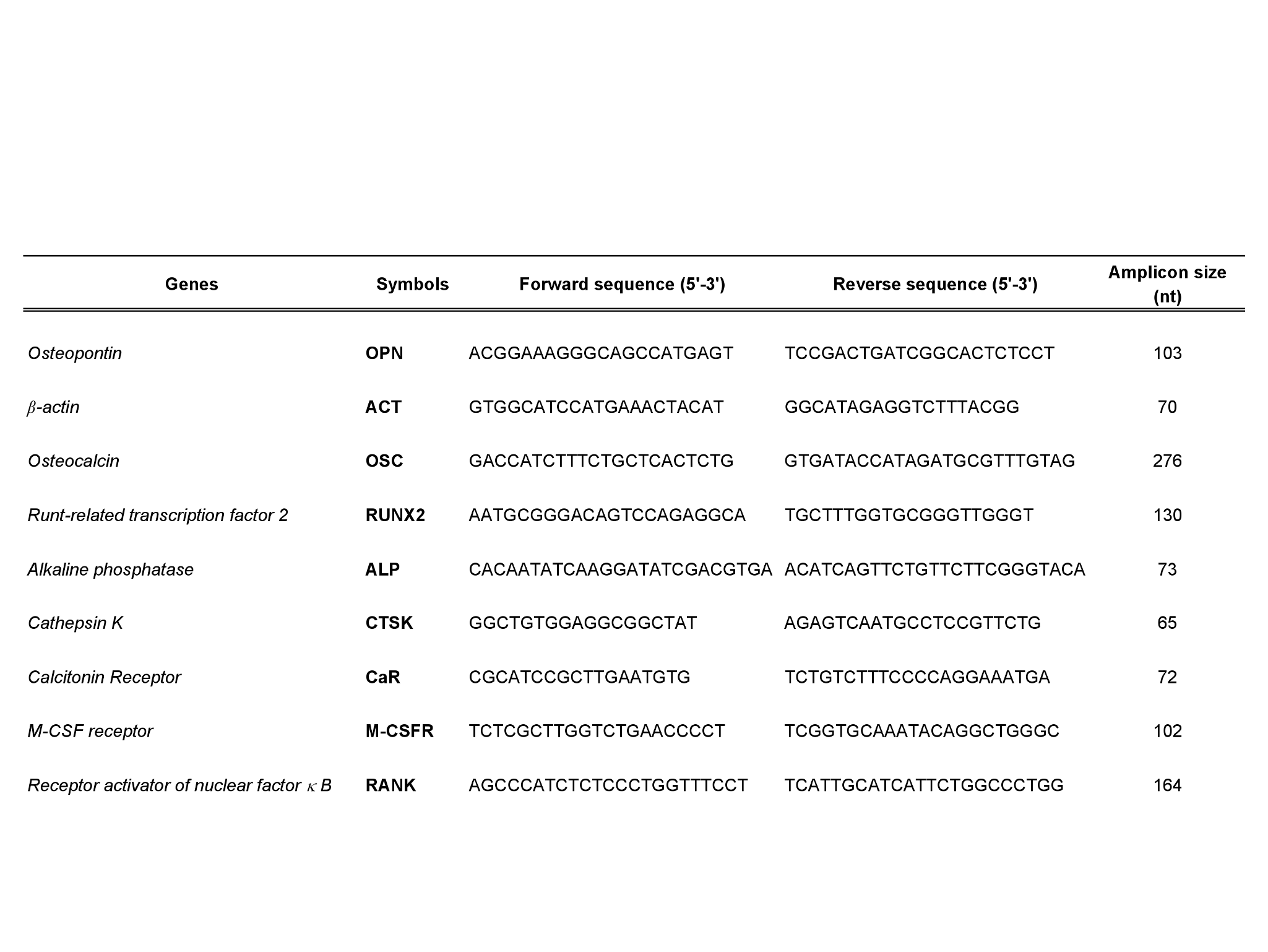

Supplement: Table S1 — Primer sequence list. (TIF) [file pone.0030788.s006.tif]

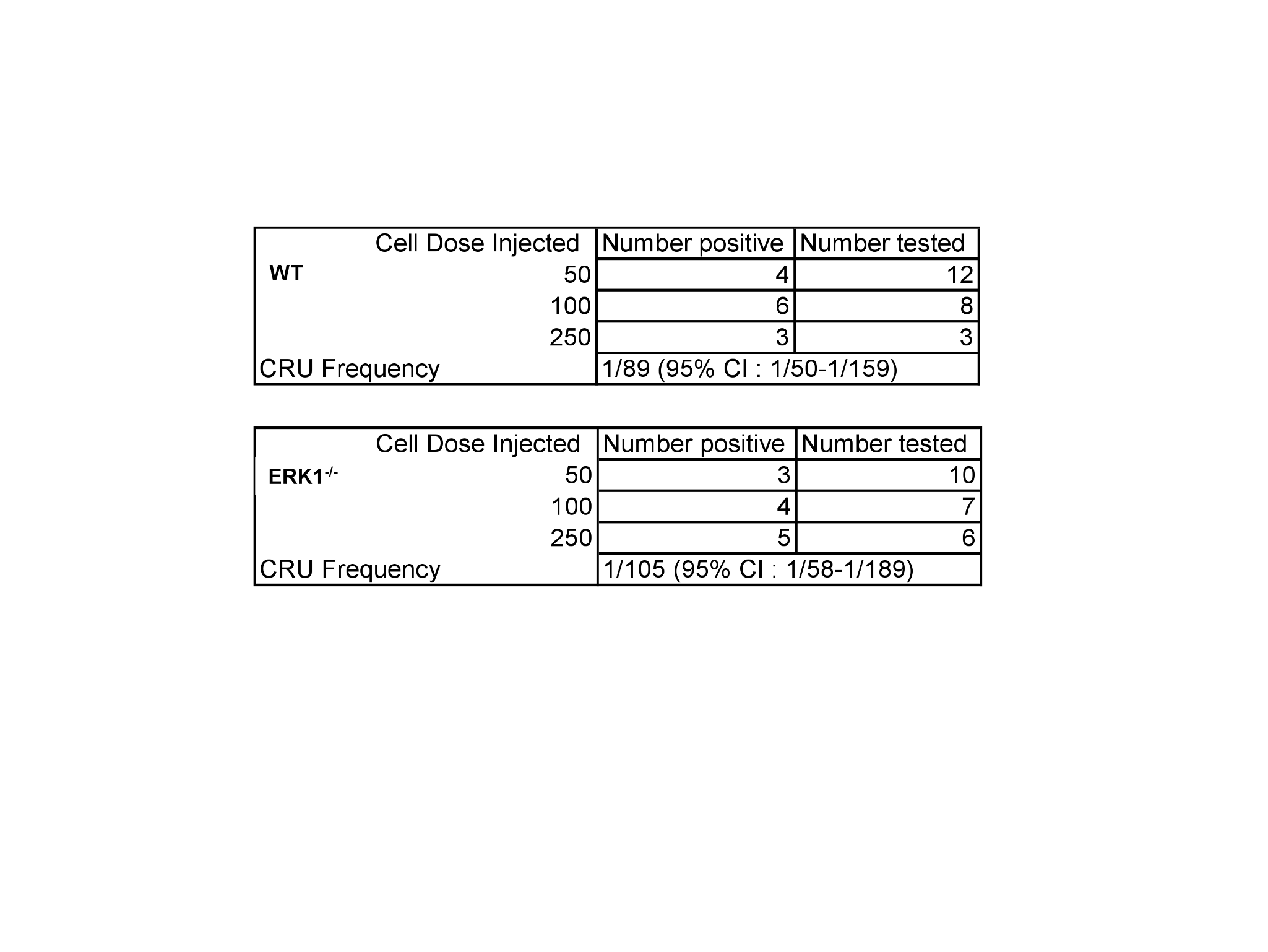

Supplement: Table S2 — CRU frequencies of WT and ERK1−/− HSCs. (TIF) [file pone.0030788.s007.tif]
